# Characterizing the use of virtual care in primary care settings during the COVID-19 pandemic: a retrospective cohort study

**DOI:** 10.1186/s12875-022-01890-w

**Published:** 2022-12-10

**Authors:** Alexander Singer, Leanne Kosowan, Lisa LaBine, Daniel Shenoda, Alan Katz, Elissa M Abrams, Gayle Halas, Sabrina T. Wong, Siddhesh Talpade, Sarah Kirby, Alanna Baldwin, Jose Francois

**Affiliations:** 1 Department of Family Medicine, Rady Faculty of Health Sciences, University of Manitoba, D009-780 Bannatyne Ave, MB R3T2N2 Winnipeg, Canada; 2 Departments of Community Health Science and Family Medicine, Rady Faculty of Health Sciences, University of Manitoba, MB Winnipeg, Canada; 3 Manitoba Centre for Health Policy, Winnipeg, MB Canada; 4 Department of Pediatrics, Section of Allergy and Clinical Immunology, Rady Faculty of Health Sciences, University of Manitoba, Winnipeg, MB Canada; 5 Department of Pediatrics, Division of Allergy and Immunology, University of British Columbia, Vancouver, BC Canada; 6 School of Nursing and Centre for Health Services and Policy Research, University of British Columbia, Vancouver, BC Canada; 7 Planning and Knowledge Management, Manitoba Health and Seniors Care, Winnipeg, MB Canada; 8 George and Fay Yee Centre for Healthcare Innovation, University of Manitoba, MB Winnipeg, Canada

**Keywords:** Primary health care, COVID-19, Telemedicine, Virtual care, Medical informatics, Health care quality, access, evaluation

## Abstract

**Background:**

In March 2020, Canada implemented restrictions to curb viral transmission of COVID-19, which resulted in abrupt disruptions to conventional (in-person) clinical care. To retain continuity of care the delivery of primary care services shifted to virtual care. This study examined the nature of virtual visits, characterizing the use and users of virtual care in primary care settings from March 14/20 to June 30/20 of the COVID-19 pandemic.

**Methods:**

Retrospective cohort study of primary care providers in Manitoba, Canada that participate in the Manitoba Primary Care Research Network (MaPCReN) and offered ≥ 1 virtual care visit between 03/14/20 and 06/30/20 representing 142,616 patients. Tariff codes from billing records determined the visit type (clinic visit, virtual care). Between 03/14/20, and 06/30/20, we assessed each visit for a follow-up visit between the same patient and provider for the same diagnosis code. Patient (sex, age, comorbidities, visit frequency, prescriptions) and provider (sex, age, clinic location, provider type, remuneration, country of graduation, return visit rate) characteristics describe the study population by visit type. Generalized estimating equation models describe factors associated with virtual care.

**Results:**

There were 146,372 visits provided by 154 primary care providers between 03/14/20 and 06/30/20, of which 33.6% were virtual care. Female patients (OR 1.16, CI 1.09–1.22), patients with ≥ 3 comorbidities (OR 1.71, CI 1.44–2.02), and patients with ≥ 10 prescriptions (OR 2.71, 2.2–1.53) had higher odds of receiving at least one virtual care visit compared to male patients, patients with no comorbidities and patients with no prescriptions. There was no significant difference between the number of follow-up visits that were provided as a clinic visit compared to a virtual care visit (8.7% vs. 5.8%) (*p* = 0.6496).

**Conclusion:**

Early in the pandemic restrictions, approximately one-third of visits were virtual. Virtual care was utilized by patients with more comorbidities and prescriptions, suggesting that patients with chronic disease requiring ongoing care utilized virtual care. Virtual care as a primary care visit type continues to evolve. Ongoing provision of virtual care can enhance quality, patient-centered care moving forward.

**Supplementary Information:**

The online version contains supplementary material available at 10.1186/s12875-022-01890-w.

## Background

Virtual care is defined as any interaction between patients and members of their circle of care, occurring remotely, using any form of communication or information technologies, to facilitate or maximize patient care [1]. Virtual care has been used in Canada for delivery of primary care since the 1970s, although utilization was relatively low [1–5]. A 2018 survey for the Canadian Medical Association reported that less than 8% of health care delivery in Canada was delivered virtually and only 4% of primary care physicians offered virtual care services [3, 4].

In Canada, the delivery of universal health care is a provincial/territorial responsibility and primary care is delivered through independently operated community-based clinics that typically represent the first point of contact with the health system. In Manitoba, primary care providers submit tariff (procedure) codes and a single International Classification of Disease, ninth revision, clinical modification (ICD-9) code following each patient visit to the provincial health insurance system for remuneration. Alternate funded (salaried) physicians and nurse practitioners are also required to submit tariff and ICD-9 codes in an identical manner for administrative purposes although they are not directly tied to payment. Prior to the COVID-19 pandemic, only three Canadian provinces – British Columbia, Alberta and Ontario – offered reimbursement for select virtual care services [6–9]. Within primary care, there was very limited integration of virtual care in clinical practice, and it was considered more of a complement to existing modes of care [2, 5, 8, 10].

The COVID-19 pandemic has resulted in abrupt disruptions to conventional (in-person) clinical care and highlighted the need for other models of care such as virtual care to meet patient needs [11]. In an attempt to facilitate safe access to health care for patients, while also curbing viral transmission associated with in-person clinical care, many primary care services quickly implemented virtual care [8, 12–15]. In late March of 2020, the province of Manitoba introduced virtual care tariffs, with retroactive implementation to March 14, 2020, for use during the pandemic to reduce in-person clinical visits and support virtual care visits [11, 16].

As a result of the pandemic, the adoption of virtual care for health services was both accelerated and widespread. Some early studies examined virtual care during the first months of the pandemic, including one from the US by Ferguson et al. (2021) that reported a 44% increase in the number of primary care visits provided virtually within Veteran Affairs. Veterans with lower income, higher disability and more chronic conditions were more likely to receive virtual care [17]. It has been suggested that virtual care can improve access to health care services particularly for patients in remote locations or among patients with health conditions limiting their mobility [8]. In addition, virtual care may be convenient for both patients and providers, and promote continuity of care by ensuring patients connect with their provider [6, 8]. However, virtual care may negatively impact care by creating a digital divide that limits access to virtual care services among patients with poor internet access or discomfort with technology including patients with lower income, lower education levels, immigrants, older adults and those living in rural settings [6, 8]. Heyworth et al. (2020) suggest the pandemic has been monumental in driving utilization of virtual care for health services and emphasized an emerging need to better understand the optimal combination of virtual and in-person clinical care for diverse conditions and patient populations [18].

As the pandemic continues to unfold, primary care providers need to adapt their practices to public health recommendations provided by the Canadian government. The accelerated and widespread adoption of virtual care requires a comprehensive investigation of how it has been used, to provide a better understanding that will support its sustained use as a mechanism for primary health care delivery in response to and following the COVID-19 pandemic [17, 18]. Focused on the first four months of the pandemic, this study explores the patient and provider factors that influenced the use of virtual care in primary care settings in Manitoba.

## Methods

### Aim

This study describes use of virtual care amongst primary care providers to provide direct patient care (visits) during the first wave of the COVID-19 pandemic in Manitoba, Canada. In addition, we assess patient and provider characteristics that are associated with virtual care use.

### Setting

This retrospective cohort study used data generated by the Manitoba Primary Care Research Network (MaPCReN), a practice-based research network within the Canadian Primary Care Sentential Surveillance Network (CPCSSN) [19]. CPCSSN is the largest electronic medical record (EMR) database in Canada and currently the most extensive source of point-of-care data extracted and processed from consenting primary care providers. Participation in MaPCReN continues to expand and as of June 2021, MaPCReN included de-identified EMR data from 265 primary care providers, including family physicians, nurse practitioners and community pediatricians, providing primary care to ~ 21% of the Manitoba population. We analyzed all patient visits with a primary care provider participating in MaPCReN between January 1, 2018 and June 30, 2020 to explore the use of virtual care between March 14, 2020 and June 30, 2020.

The study was approved by the Health Research Ethics Board at the University of Manitoba (File No. HS24158).

### Cohort inclusion criteria

**Fig. 1:**
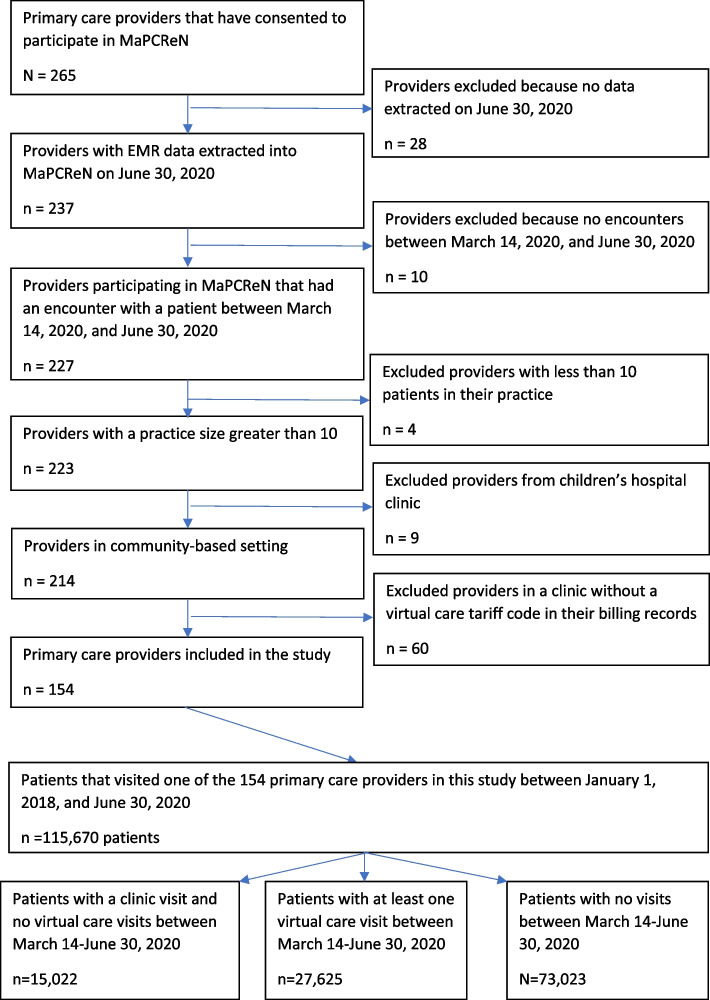
Flow diagram of providers and patients participants

We included primary care providers with at least one virtual care tariff code between March 14, 2020 and June 30, 2020. Providers with a practice size < 10 patients as of June 30, 2020 and providers from a hospital clinic were not included within this study. Hospital-based care settings received different directions for the use of virtual care services during the pandemic. Providers working at a clinic that did not report any virtual tariff codes in the billing record (MaPCReN data source) between March 14, 2020 and June 30, 2020 were not included in the final analysis (Fig. 1) (Supp. Table describes the patient cohort). 

### Data definitions

The main outcome of interest is whether an individual had a virtual visit with a primary care provider. We classified each visit with a MaPCReN participating primary care provider as ‘in-person’ or ‘virtual care’ based on the tariff code(s) recorded in the EMR. Virtual care visits represent any interactions between a primary care provider and a patient occurring by telephone call or video-conference technology. Patients with at least one visit to one of the 154 primary care providers participating in this study (Fig. 1) between January 1, 2018 and June 30, 2020 were included. Patients were categorized based on their visits between March 14, 2020 to June 30, 2020 as having had only a clinic visit (in-person), at least one virtual care visit or no visit or interaction of any kind (Fig. 1).

To examine factors that may be associated with virtual care visits, a number of patient and provider-level covariates were considered. Covariates included patient sex (as reported in the EMR), age, number of comorbidities, number of prescribed medications and number of annual visits of any form with their primary care provider. Patient age was determined on June 30, 2020. Co-morbidities were identified using the most current validated case definitions from CPCSSN (i.e., depression, diabetes mellitus, hypertension, chronic obstructive pulmonary disease, osteoarthritis, dementia, Parkinson’s disease, epilepsy [20], and chronic kidney disease [21]). The number of comorbidities of a patient was categorized as 0, 1–2, ≥ 3. Patients with ≥ 3 comorbidities are shown to have higher health care needs [22]. We identified medications using the Anatomical Therapeutic Chemical (ATC) code. We calculated the annual average number of prescribed medications for each patient in the previous two years (i.e., 2018, 2019) and categorized patients based on the average number of annual prescribed medications: 0 medications, 1–4 medications, 5–9 medications, and 10 or more. Polypharmacy (5–9 medications) and hyper-polypharmacy (≥ 10 medications) has been shown to be associated with poorer health outcomes [23]. Patients were categorized based on the average number of annual visits in the previous two years (i.e., 2018, 2019) representing their visit frequency: 0–2, 3–5, 6–9, and 10 or more visits. Previous research has classified ≥ 10 visits annually as high use of primary care services [24].

Provider covariates included sex, age, medical school location, clinician type, funding model and return visit rate. Provider age was determined as of June 30, 2020. Medical school location was dichotomised as Canadian or international medical school graduation. Clinician type included family physician, nurse practitioner, or community pediatrician. Funding model was categorized as fee-for-service or alternative funding. Fee-for-service described providers who receive remuneration based on submitted tariff codes. Return visit rate is used as a measure for appropriate access to primary care and as a gauge for clinic and health system planning. Return visit rate provided the expected number of visits per year, per eligible patient in the practice and was determined by dividing the annual visit frequency by the number of patients in the practice. Annual return visit rate for 2020 was derived by multiplying the actual rate between March 1, 2020 and June 30, 2020 by three.

### Statistical analysis

We describe the study population using means (standard deviation (SD)), medians (interquartile range (IQR)), and frequencies (percent). We compared patient characteristics by visit type (i.e. clinic visit, at least one virtual care visit, and no visit) using t-tests or chi-square tests, as appropriate. Between March 14, 2020, and June 30, 2020, we assessed each visit for a follow-up visit between the same patient and provider for the same ICD-9 code, and also compared the 2020 annual visit rate to the annual visit rate in 2019 for each provider.

For patients with a visit between March 14, 2020 and June 30, 2020, a multivariate logistic regression model using generalized estimating equations assessed the association between patients with at least one virtual care visit (yes vs. no) and characteristics of the patient (≥ 18 vs. ≥60, 19–39 vs. ≥60, 40–59 vs. ≥60), sex (female vs. male), number of comorbidities (≥ 3 vs. 0, 1–2 vs.0), annual number of prescriptions (1–4 vs. 0, 5–9 vs. 0, ≥ 10 vs. 0), and annual visit rate (continuous)) and provider (sex (female vs. male), age (less than mean age (47.2 years) vs. ≥ mean age) remuneration (alternative funding vs. fee-for-service), medical training (Canadian graduate vs. international graduate), and clinic location (rural vs. urban clinic). We used a generalized estimating equation approach to account for the clustering of patients within a provider’s practice. We also performed a sensitivity analysis to test associations that might be attributed to providers practicing at the same clinic, which did not significantly change the results. We report associations using the adjusted odds ratios (aOR) with 95% confidence intervals (CI). Statistical analyses were performed using SAS V9.4(SAS Institute Inc. Cary, NC).

## Results

Among the 154 primary care providers in this study, the majority (79%) were family physicians, 14% were nurse practitioners and 7% were community-based pediatricians. 71% provided care at an urban clinic, 63% were female, 34% were fee-for-service, and 88% graduated from a Canadian medical school.

Between March 14, 2020, and June 30, 2020, there were 146,372 visits with a participating provider and of these, 33.6% (*n* = 49,118) were virtual care visits. There were 149/154 (96.8%) providers that provided at least one visit as a virtual care visit. An estimated annual *return* visit rate was calculated based on quarterly time segments from 2019 to 2020. The rates were then compared using t-test to demonstrate the estimated return visit rate for 2020 (median 4.0 (IQR 2.2)) was not significantly different than the 2019 return visit rate (median 4.1 (IQR1.8)) (*p* = 0.6627). (Table 1)

**Table 1:** Characteristics of MaPCReN primary care providers who met cohort inclusion criteria (*n*= 154)

Variable	Primary Care Providers
Female (vs. male) provider, n (%)	97 (63.0%)
Provider age, mean (SD)	47.2 years (1.0)
Urban (vs. rural) clinic, n (%)	109 (70.8%)
Canadian medical school graduate (vs. international graduate), n (%)	136 (88.3%)
Family physician (vs. nurse practitioner/community pediatrician), n (%)	122 (79.2%)
Fee-For-Service (vs. alternative funding) provider, n (%)	52 (33.8%)
Practice size, median (IQR)	735 patients (634)
Return Visit Rate 2019 per provider, median (IQR)	4.1 visits (1.8)
Estimated Return Visit Rate 2020 per provider, median (IQR)^a^	4.0 visits (2.2)

^a^Estimate calculated as one quarter (March 1-June 30, 2020) multiplied by 3

In unadjusted comparisons, patients with no visits were younger (mean age of 38.0 years (SD 24.9)) than patients with a virtual care visit (48.7 years (SD 23.7)) (*p* = 0.0001) and had lower annual visit frequency in the previous 2 years, with 45.2% having less than 2 visits in the previous years compared to patients with a virtual care (13.3%) or clinic visit (22.4%). Among patients with greater health care utilization, 22.7% of patients with a virtual care visit had 10 or more visits with their primary care provider in the previous two years compared to patients with a clinic visit (14.9%, *p* = < 0.0001) or no visit (4.2%, *p* = < 0.0001) (Table 2).

Patients with no visits were significantly more likely to have no chronic diseases (66.2%) and no prescribed medications in the previous 2 years (49.4%) compared to patients with a virtual care appointment (32.5%, 7.8%, respectively) or clinic visit (49.7%, 19.4%), respectively. Patients with at least one virtual care visit were older than patients with a clinic visit (48.7 years (SD23.6) vs. 40.7 years (SD25.5), *p* = < 0.01), were more likely to have three or more chronic diseases (10.4% vs. 5.5%, *p* = < 0.0001) and have 10 or more prescribed medications (28.4% vs. 17.2%, *p* = < 0.0001) (Table 2).

**Table 2:** Characteristics of patients who visited a primary care provider between March 14, 2020 and June 30, 2020, by visit type *n*= 115,670

Variable	Patients with a clinic visit and no virtual care visit *n* = 15,022	Patients with at least one virtual care visit *n* = 27,625	Patients with no visits *n* = 73,023
Female (vs. male) patients, n (%)	8271 (55.1%)	16,562 (60.0%)	37,707 (51.7%)
Patient age, mean (SD)	40.7 (25.5)	48.7 (23.7)	38.0 (24.9)
Patient age, n (%)			
≤ 18 years	3691 (24.6%)	3709 (13.4%)	20,565 (28.2%)
19–39 years	3594 (23.9%)	6121 (22.2%)	19,891 (27.2%)
40–59 years	3572 (23.8%)	7318 (26.5%)	16,104 (22.1%)
60 + years	4165 (27.7%)	10,477 (37.9%)	16,463 (22.5%)
Annual visit frequency, n (%)			
< 2 visits	3364 (22.4%)	3679 (13.3%)	32,968 (45.2%)
2–5 visits	5462 (36.4%)	8750 (31.7%)	27,518 (37.7%)
5–10 visits	3960 (26.4%)	8936 (32.4%)	9504 (13.0%)
≥ 10 visits	2236 (14.9%)	6260 (22.7%)	3033 (4.2%)
Number of comorbidities, n (%)			
0	7471 (49.7%)	8974 (32.5%)	48,350 (66.2%)
1–2	6733 (44.8%)	15,787 (57.2%)	22,597 (31.0%)
≥3	818 (5.5%)	2864 (10.4%)	2076 (2.8%)
Annual number of prescriptions, n (%)			
0 medications	2919 (19.4%)	2148 (7.8%)	36,066 (49.4%)
1–4 medications	7087 (47.2%)	11,516 (41.7%)	29,760 (40.8%)
5–9 medications	2431 (16.2%)	6104 (22.1%)	4539 (6.2%)
≥10 medications	2585 (17.2%)	7857 (28.4%)	2658 (3.6%)

Patients with a greater number of diagnosed comorbidities or higher annual number of prescribed medications had significantly higher odds of having a virtual care visit. Patients aged less than 19 years, 19–39 years and 40–59 years were significantly less likely to have a virtual care visit compared to patients aged 60 years or older (Table 3).

**Table 3:** Patient and provider characteristics associated with having a virtual care visit

Provider variables (*n* = 154 providers)	Adjusted OR^a,b^	95% Confidence Interval
Female vs. male provider	1.16	0.92–1.48
Provider age (less than 47.2 years vs. greater than 47.2 years)	0.87	0.68–1.12
Canadian graduate vs. international graduate	1.34	0.83–2.18
Alternative funding vs. fee-for-service	0.81	0.57–1.16
Rural vs. urban clinic	1.21	0.8–1.82
Patient variables (*n* = 42,647 patients)	OR	95% Confidence Interval
Female vs. male patient	**1.14**	**1.08–1.21**
≥ 3 comorbidities vs. no comorbidities	**1.78**	**1.51–2.09**
1–2 comorbidities vs. no comorbidities	**1.41**	**1.28–1.55**
≥ 10 prescriptions vs. no prescriptions	**2.70**	**2.22–3.29**
5–9 prescriptions vs. no prescriptions	**2.42**	**2.07–2.85**
1–4 prescriptions vs. no prescriptions	**1.86**	**1.68–2.07**
Patient age (≤ 18 years vs. ≥60 years)	**0.69**	**0.58–0.83**
Patient age (19–39 years vs. ≥60 years)	**0.89**	**0.81–0.98**
Patient age (40–59 years vs. ≥60 years)	**0.94**	**0.86–1.03**
Annual visit frequency (per 1 visit increase)	1.00	0.99-1.00

^a^Multivariate logistic regression model using generalized estimating equations to assess the association between patients with at least one virtual care visit (yes vs. no)

^b^Bolding indicates statistical significance

We also examined the data to determine if follow up visits were more frequently occurring after a virtual visit versus after an in-person clinic visit. A chi-square test demonstrated no significant difference (*p* = 0.6496) in the occurrence of follow up visits (i.e. patient receiving a second appointment for the same ICD-9 code) between those who had a clinic visit (*n* = 5654, 8.7%) and those who had a virtual visit (*n* = 3829, 5.8%). Among patients who had a follow-up appointment, for 55.9% (*n* = 13,931 visits) the visit type did not change at the follow-up visit. Among patients that changed visit type, 42.2% (*n* = 4639 visits) had an initial in-person visit and their follow-up visit was virtual care. In comparison, 26.9% (*n* = 2957 visits) of virtual care visits changed to an in-person appointment for their follow-up appointment.

## Discussion

Our study describes the use and users of virtual care in primary care settings during the first wave of the COVID-19 pandemic and identifies patient- and provider-level factors associated with its use. During the first four months of the COVID-19 pandemic (i.e., March – June 2020), Manitoba primary care settings saw an increase in the use of virtual care services, with virtual care accounting for 33% of primary care visits. Our results suggest that many primary care practices were able to transition to some form of virtual visit(s) to provide care in the initial months of the pandemic.

Patients with the most comorbidities and highest annual number of prescriptions were more likely to have at least one virtual care visit, indicating that those who required ongoing attention from primary care made use of virtual care services. Patients requiring continuity of care for the management of chronic disease had greater likelihood of accessing care virtually at least one time during the study period. Further to this the literature suggests virtual care provides improved access among patients with chronic conditions [6, 8]. Ideal chronic disease management often requires continuity with health provider(s), and patients with chronic illness are more likely to have new or changing health concerns, which likely explains this finding. Virtual care can promote continuity of care, ensuring patients can access health services from their primary care provider in a manner that is convenient for both, while still appropriately managing health conditions [6, 8, 25]. Lending further support, we found that patients who were seen virtually at least one time were more likely to receive a follow-up visit that was also virtual, implying that care was needed on an ongoing basis. Several studies have similarly demonstrated that virtual care services helped health care providers adhere to physical distancing and other public health measures intended to control the spread of COVID-19 [6, 25–28] and may have even improved access to care for patients in remote locations, or with mobility and other health conditions that rendered clinic visits difficult or unsafe [6, 8, 25].

Our results also indicated that patients with only in-person visits (no virtual care) had more chronic diseases and prescriptions than patients without any visits to a primary care provider. These findings are consistent with other studies, where individuals with greater health needs or comorbidities continued to access primary care services during the pandemic, despite a decrease in the frequency of visits overall [29–32].

As primary health care providers learn how best to integrate virtual care into their practice, other studies, particularly in areas such as mental health, have reported quality of care and outcomes regardless of in-person or virtual formats [1, 33, 34]. Further research is needed to better understand the effectiveness of virtual care for the broad range of conditions managed in primary care settings and will remain important as the use of virtual care evolves throughout and beyond the pandemic.

A key strength of this study is that it provides a clear description of the initial uptake of virtual care in Manitoba’s primary care settings. Data trends seen here are likely similar to other clinical settings where funding models rapidly shifted to accommodate physical distancing and compliance with other public health restrictions. In Canada, provinces and territories implemented virtual care tariff/billing codes for synchronous visits along a comparable timeline [35]. Exploring both patient and provider characteristics associated with visit type offers opportunities to learn from the rapid shift to virtual models of care to better inform future changes to health care delivery.

This study also has several limitations. While the study cohort included a large number of patients and providers from various practice types and locations, it is uncertain if our cohort is representative of other jurisdictions. It is possible our study did not capture some virtual or in-person care that was not remunerated since we relied on documented tariff codes to assign virtual or in-person care. Similarly, fee-for-service providers were more likely to work at a clinic that was not included because there were no virtual care tariff codes in their billing records. Future research will assist in analyzing virtual care provided by a larger number of fee-for-service providers. Additionally, there may be patient or provider characteristics that could not be measured due to limitations in the database and may have contributed to the use of virtual care services. For example, we have focused on health conditions with a validated case definition, which limited the number of conditions identified in the EMR data. Further, not all patients have a dedicated primary care provider, so those not regularly seeing one (e.g., relying on tertiary services) would not be included in our analysis. Lastly, our findings cover only the early pandemic period and thus represent care provided by early adopters of virtual care. Our return visit rate is based on care seeking behaviors in the first four months of the pandemic, which may not accurately portray changes in care seeking behavior through the full year.

Our study adds to the literature, describing the substantial shift to virtual care services and changes to primary care delivery in the first four months of the COVID-19 pandemic. We found that patients requiring health care services during the first four months of the pandemic received care. There was a positive association between the use of virtual care and patient health care needs. While these changes were in response to an unexpected crisis, the adoption of virtual health care during this time demonstrates that significant change can be rapidly implemented with the necessary drivers. Future research should focus on the impact and ongoing use of virtual care on patients, provider workload, quality of care, clinic workflow, provider and patient satisfaction with health services as well as factors that facilitated the timely adoption and access to virtual care services in a clinical setting [36, 6, 24, 37–39].

## Conclusion

The COVID-19 pandemic precipitated a shift to virtual health care services to accommodate public health recommendations while retaining continuity of primary care services. This study describes changes in the provision of primary care that occurred prior to and within the initial months of the pandemic, including patient and provider factors significantly associated with virtual care utilization. As virtual health services continue to evolve, understanding the drivers, barriers, and nuances of technology-enabled health care will help inform policy and governance, as well as promote virtual care service options that enhance equitable, quality, patient-centred care.

## Supplementary Information

**Additional file 1:** **Supp Table.** Characteristics of study cohort *n*=115,670.

## Data Availability

The datasets generated and/or analysed during the current study are not publicly available due to the confidential nature of the data governed by PHIA legislation but are available from the corresponding author on reasonable request.

## Abbreviations

COVID-19: Coronavirus Disease 2019
MaPCReN: Manitoba Primary Care Research Network
CPCSSN: Canadian Primary Care Sentential Surveillance Network
EMR: Electronic Medical Record
ICD-9: International Classification of Disease, ninth revision, clinical medication
ATC: Anatomical Therapeutic Chemical code
SD: Standard deviation
IQR: Interquartile range
OR: Odds ratio
CI: 95% confidence interval
