# Supplementary material for: Characterizing the use of virtual care in primary care settings during the COVID-19 pandemic: a retrospective cohort study
Source: BMC Prim Care. 2022 Dec 10;23:320. doi: 10.1186/s12875-022-01890-w (PMC9736717; doi:10.1186/s12875-022-01890-w)
Supplement: Supplementary file 1 — Additional file 1: Supp Table. Characteristics of study cohort n=115,670. [file 12875_2022_1890_MOESM1_ESM.docx]

| **Supp Table: Characteristics of patients included in the study**  **n=115,670** | |
| --- | --- |
| Variable |  |
| Female (vs male) patients, n (%) | 62,540(54.1%) |
| Patient age (mean, SD) | 41.4(25.1) |
| Patient age, n (%) | |
| ≤18 years | 27,965(24.2%) |
| 19-39 years | 29,606(25.6%) |
| 40-59 years | 26,994(23.3%) |
| 60+ years | 31,105(26.9%) |
| Annual visit frequency, n (%) | |
| <2 visits | 40,011(34.6%) |
| 2-5 visits | 41,730(36.1%) |
| 5-10 visits | 22,400(19.4%) |
| ≥10 visits | 11,529(10.0%) |
| Number of comorbidities, n (%) | |
| 0 | 64,795(56.0%) |
| 1-2 | 45,117(39.0%) |
| >3 | 5,758(5.0%) |
| Annual number of prescriptions, n(%) | |
| 0 medications | 41,133(35.6%) |
| 1-4 medications | 48,363(41.8%) |
| 5-9 medications | 13,074(11.3%) |
| >10 medications | 13,100(11.3%) |
|  | |
